# Anthocyanins encapsulated by PLGA@PEG nanoparticles potentially improved its free radical scavenging capabilities via p38/JNK pathway against Aβ_1–42_-induced oxidative stress

**DOI:** 10.1186/s12951-016-0227-4

**Published:** 2017-02-07

**Authors:** Faiz Ul Amin, Shahid Ali Shah, Haroon Badshah, Mehtab Khan, Myeong Ok Kim

**Affiliations:** Department of Biology and Applied Life Science (BK 21), College of Natural Sciences Gyeongsang National University, Jinju, 660-701 South Korea

**Keywords:** Alzheimer’s disease, PLGA@PEG-nanoparticles, Anthocyanins, Oxidative stress, Neuroprotection

## Abstract

**Background:**

In order to increase the bioavailability of hydrophilic unstable drugs like anthocyanins, we employed a polymer-based nanoparticles approach due to its unique properties such as high stability, improved bioavailability and high water-soluble drug loading efficiency. Anthocyanins constitute a subfamily of flavonoids that possess anti-oxidative, anti-inflammatory and neuroprotective properties. However, anthocyanins are unstable because their phenolic hydroxyl groups are easily oxidized into quinones, causing a reduced biological activity. To overcome this drawback and improve the free radical scavenging capabilities of anthocyanins, in the current study we for the first time encapsulated the anthocyanins in biodegradable nanoparticle formulation based on poly (lactide-*co*-glycolide) (PLGA) and a stabilizer polyethylene glycol (PEG)-2000. The biological activity and neuroprotective effect of anthocyanin loaded nanoparticles (An-NPs) were investigated in SH-SY5Y cell lines.

**Results:**

Morphological examination under transmission electron microscopy (TEM) showed the formation of smooth spherically shaped nanoparticles. The average particle size and zeta potential of An-NPs were in the range of 120–165 nm and −12 mV respectively, with a low polydispersity index (0.4) and displayed a biphasic release profile in vitro. Anthocyanins encapsulation in PLGA@PEG nanoparticles (NPs) did not destroy its inherent properties and exhibit more potent neuroprotective properties. An-NPs were nontoxic to SH-SY5Y cells and increased their cell viability against Aβ_1–42_ by its free radical scavenging characteristics and abrogated ROS generation via the p38-MAPK/JNK pathways accompanied by induction of endogenous nuclear factor erythroid 2-related factor 2 (Nrf2) and heme oxygenase 1 (HO-1). Comparative to native bulk anthocyanins, An-NPs effectively attenuated Alzheimer’s markers like APP (amyloid precursor protein), BACE-1 (beta-site amyloid precursor protein cleaving enzyme 1), neuroinflammatory markers such as p-NF-kB (phospho-nuclear factor kappa B), TNF-α (tumor necrosis factor) and iNOS (inducible nitric oxide synthase) and neuroapoptotic markers including Bax, Bcl_2_, and Caspase-3 protein expressions accompanied by neurodegeneration against Aβ_1–42_ in SH-SY5Y cell lines.

**Conclusions:**

Overall, this data not only confirmed the therapeutic potential of anthocyanins in reducing AD pathology but also offer an effective way to improve the efficiency of anthocyanins through the use of nanodrug delivery systems.

**Electronic supplementary material:**

The online version of this article (doi:10.1186/s12951-016-0227-4) contains supplementary material, which is available to authorized users.

## Background

A variety of chemical drugs have discovered and developed over the past several decades, but a few problems such as fast elimination and denaturation or degradation are still remain to be determined [1]. Many attempts to solve these problems have been made by using high dose or multi-treatment of the drugs. However, it could be a very dangerous choice for efficient therapy, because if overdoses out of range of therapeutic windows are used [2] nonspecific toxicity of drugs could be caused [3]. One approach to overcome these problems was the packaging of the drugs into a particulate carrier system, i.e. solid polymeric nanoparticles and lipidic systems such as oil-in-water (O/W) emulsions and the liposomes [4]. In general, high drug stability in drug delivery technology leads to enhance the bioavailability of drug [5]. Incorporation of the drug into a particulate carrier protects it against the outer stresses in vitro and in vivo [6] maintain long-term circulation [7] and design the delivery to target site [8]. Poly (lactic-co-glycolic acid) (PLGA) is one of the most successfully used biodegradable polymers because its hydrolysis leads to metabolite monomers, lactic acid and glycolic acid. These two monomers are endogenous and easily metabolized by the body via the Krebs cycle; a minimal systemic toxicity is associated with the use of PLGA for drug delivery or biomaterial applications [9]. PLGA is approved by the US FDA and European Medicine Agency (EMA) in various drug delivery systems in humans. The polymers are commercially available with different molecular weights and copolymer compositions. The degradation time can vary from several months to several years, depending on the molecular weight and copolymer [10, 11]. The forms of PLGA are usually identified by the monomers ratio used. For example, PLGA 50:50 identifies a copolymer whose composition is 50% lactic acid and 50% glycolic acid. Poly (lactic acid) (PLA) has also been used to a lesser extent than PLGA due to the lower degradation rate [12].

The surface modification of a polymer with nontoxic and blood compatible material is essential in order to avoid recognition by macrophages, to prolong blood circulation time and sustained release of the encapsulated drugs [13, 14]. Poly (ethylene glycol) (PEG) is widely used as hydrophilic nontoxic segment in combination with hydrophobic biodegradable aliphatic polyesters [15–18]. Incorporation of a hydrophilic PEG group on the surface of nanoparticles was found to show resistance against opsonization and phagocytosis and showed prolonged residence time in blood compared to the nanoparticles prepared without PEG [15, 16, 18].

Alzheimer’s disease (AD) is the most common age-related neurodegenerative disorder characterised by progressive learning and memory deficit. The amyloid hypothesis of AD postulates that β-amyloid (Aβ) deposition and neurotoxicity play a causative role in AD [19]. The Aβ_1–42_ is neurotoxic both in vitro and in vivo model [20, 21]. Recent evidence suggests that the neurotoxic properties of Aβ are mediated by oxidative stress [22].

Importantly nuclear factor erythroid 2-related factor 2 (Nrf2) is a key redox-regulated gene that has a critical role against oxidative stress, Nrf2 nuclear level decreased in the hippocampus of AD patients [23]. Nrf2 regulated the several endogenous redox-regulated enzymes such as heme oxygenase-1 (HO-1) and glutathione cysteine ligase modulatory subunit (GCLM). Notably, heme oxygenase-1 (HO-1) is beneficial in various diseases, especially neurodegenerative diseases such as AD [24]. Recently, investigated that nuclear translocation of Nrf-2 increased the expression of HO-1 [25]. Elevated expression of Nrf-2 both in vitro and in vivo AD model decreased the Aβ-induced neurodegeneration and oxidative stress [26].

In AD brain, activation of the MAPK pathways has been demonstrated in neurons and dystrophic neurites: c-Jun N-terminal kinase (JNK) [27, 28] and p38 [29]. Inhibition of the JNK pathway significantly reduced the toxicity attributable to Aβ in both of the studies. Increased p38 activity has been reported after Aβ treatment of microglia [30]. The downstream signal transduction of the P38 and JNK pathways has been described to activate a variety of transcription factors and generate different inflammatory mediators [31]. Furthermore, it has been described that JNK signaling induces activator protein (AP)-1-dependent BAX and caspase activation, which results in neuronal apoptosis [32].

Anthocyanins constitute a subfamily of flavonoids that possess antioxidative, anti-inflammatory, and antineurodegenerative properties [33, 34]. Anthocyanin extracted from berries can improve cognitive brain function and reduce age associated oxidative stress [35–37]. They have been shown to prevent learning and memory loss in estrogen-deficient rats [38].

In this study, we constructed anthocyanin loaded (PLGA@PEG) nanoparticle system to assess the suitability of the nanoparticles as delivery vehicles for hydrophilic drugs, and studied its release kinetics in vitro. The biological activity and neuroprotective effect of encapsulated anthocyanin were investigated in SH-SY5Y cell cultures, confirming the protection against Aβ_1–42_-induced neurotoxicity. An-NPs were more potent than native bulk anthocyanin and exhibit anti-amyloid, anti-oxidative and anti-inflammatory properties and are non-cytotoxic.

## Results

### Preparation and characterization of anthocyanins-loaded nanoparticles (An-NPs)

**Fig. 1:**
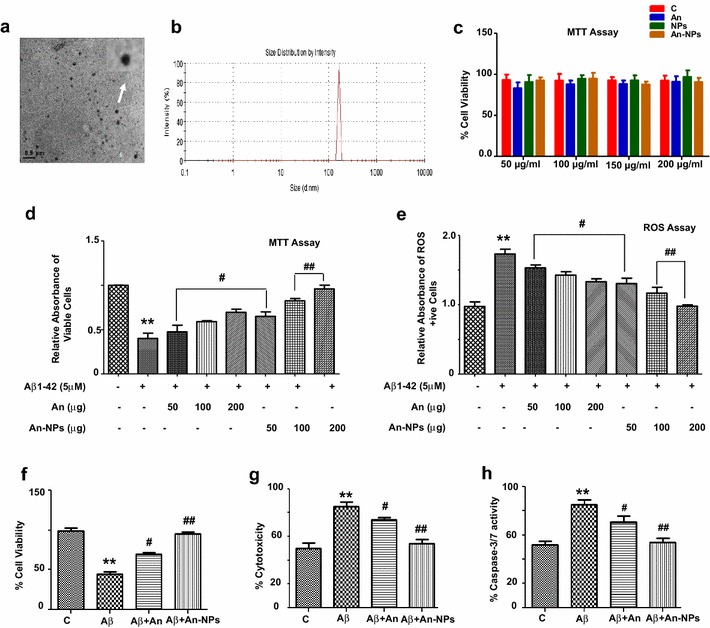
Transmission electron microscopy and DLS observations of An-NPs and its beneficial effects against Aβ_1–42_-induced neurotoxicity; **a** TEM micrograph of An-NP (*scale bar* 0.5 µm). **b** DLS analysis for the particle size of An-NPs. **c** In vitro cytotoxicity of PLGA@PEG NPs, native anthocyanin and An-NPs incubated with normal SH-SY5Y cells. Cell viability was measured by MTT assay. Four different concentrations of the test samples were added to the cells and incubated for 24 h before adding the respective assay reagents. We have observed that the nanoparticles were highly biocompatible. **d** Shown is the cell viability (MTT assay) histogram. Aβ_1–42_ (5 µM) reduced cell viability while anthocyanins and An-NPs at three different concentrations (50, 100 and 200 µg/ml) increased the cell viability of SH-SY5Y cell lines. **e** Representative ROS assay histogram. Anthocyanins and An-NPs in all three different concentrations (50, 100 and 200 µg/ml) significantly reduced Aβ_1–42_-induced (5 μM) ROS production. **e** The ApoTox-Glo Triplex Assay was performed (Promega, Promega BioSciences, LLC., San Luis Obispo, CA, USA). *Histogram* showing, cell viability. **f** Cytotoxicity and (**g**) Caspase-3/7 assays. All the related experimental details are provided in the “Methods” section. All these assays were performed in triplicate (±SEM). *Significantly different from the control; ^#^significantly different from Aβ_1–42_-treated group. Significance = **p < 0.01, ^#^p < 0.05, ^##^p < 0.01

Anthocyanins loaded PLGA@PEG nanoparticles (An-NPs) were prepared by emulsification-solvent evaporation technique. Morphological examination under transmission electron microscopy (TEM) showed the formation of smooth spherically shaped nanoparticles with an average diameter of 120–165 nm (Fig. 1a). The mean particle size and zeta potential were measured by dynamic light scattering (DLS) and Electrophoretic light scattering (ELS) analysis, respectively. The mean diameter of the NPs as determined from DLS measurement was 165 nm with a low polydispersity index (0.4), indicating the formation of almost monodispersed nanoparticles (Fig. 1b). This observation was supported by the result obtained from the morphological examination using TEM analysis (Fig. 1a). The zeta potential of the prepared NPs measured by ELS was −12 mV.

### Determination of interaction between anthocyanin and PLGA-PEG -NPs

The physical interaction between anthocyanin and NPs was determined by FT-IR (Fourier transform infrared spectroscopy) analysis. The FT-IR spectra of the An-NPs and their precursors PLGA@PEG and anthocyanin were recorded to confirm the structural composition of the final product. Additional file 1: Figure S1 shows the typical FT-IR spectra of the free anthocyanin, PLGA@PEG NPs and An-NPs. In the spectrum, no characteristics peaks can be distinguished in the An-NPs in which the characteristics absorption peaks were masked by that of NPs.

### Drug loading and in vitro drug release

Anthocyanin was employed as a model drug to evaluate the potential of polymeric NPs to encapsulate hydrophilic drugs. In order to study the bioavailability and controlled release of anthocyanin from PEGylated nanoparticles, the drug was encapsulated in the PLGA@PEG nanoparticles and its release kinetics was studied in vitro. This in vitro release profile of anthocyanin loaded PLGA@PEG nanoparticles in PBS at 37 °C is shown in Additional file 1: Figure S2. Anthocyanin was encapsulated with 60% efficiency in biodegradable nanoparticle formulation based on poly (lactide-*co*-glycolide) (PLGA) and a stabilizer polyethylene glycol (PEG)-2000, showing a biphasic release profile in vitro. All the PEGylated nanoparticles showed similar release profiles of initial burst release of drug from the nanoparticles followed by a sustained release [39]. Allen et al. [39] described that some amount of the drug may be absorbed on the surface of nanoparticles or loosely bound to the inner polymer core, which was lost during the initial stage of incubation, suggesting an initial burst release of the drug. The strongly encapsulated drug in the core domains followed slow and sustained release kinetics. On the other hand, almost all the free anthocyanin was released into the medium within 3 h (data not shown here). This result indicates that the PLGA@PEG nanoparticles effectively extended the systemic release of anthocyanin and can be used for the effective controlled delivery of hydrophilic drugs.

### Beneficial effects of An-NPs against Aβ_1–42_-induced neurotoxicity in vitro

Cytotoxicity profile of the nanoparticles was first studied in human neuroblastoma SH-SY5Y cell line using MTT assay. Four different concentrations of each of the test sample PLGA@PEG NPs, native anthocyanins and An-NPs were used for the studies. The respective assay reagents were added after incubating the cells and the nanoparticles for 24 h. From the results shown in Fig. 1c, it is concluded that An-NPs do not possess any significant cytotoxic effect and the observed cell viability was in between 85 and 95% at all mentioned concentrations. This is because both PLGA and PEG polymers are FDA approved safe materials so, these nanoparticles might be promising drug carriers with little cytotoxicity. Additionally, the same viability assays were repeated for the cytotoxic nature of An-NPs either alone or against the Aβ_1–42_-induced cytotoxicity in SH-SY5Y cell line (Fig. 1c, d). The cells received Aβ_1–42_ (5 μM) alone and in combination with three different concentrations (50, 100 and 200 µg/ml) of anthocyanin/An-NPs. The cell viability histogram reveals that Aβ_1–42_ significantly induced cell death. However, An-NPs treatment against Aβ_1–42_ significantly increased the viability (Fig. 1d). Similarly, all three different concentrations (50, 100 and 200 µg) completely inhibited Aβ_1–42-_induced ROS generation indicates that native anthocyanin and An-NPs are potent antioxidants (Fig. 1e). This also reveals that the antioxidant activity of An-NPs is more significant as compared to three different concentrations of alone anthocyanins (Fig. 1e). Furthermore, the ApoTox-Glo™ Triplex Assay (containing viability/cytotoxicity and caspase-3/7 assays) was conducted to evaluate and compare the neuroprotective role of An-NPs against Aβ_1–42_ in vitro. Here in too, treatment with An-NPs significantly reduced the neurotoxic effects of Aβ_1–42_, thereby increasing cell viability and decreasing cytotoxicity and caspase-3/7 activation. Our results also determined more significant neuroprotective effects in case of An-NPs treatment compared with alone anthocyanin**s** treated group (Fig. 1f–h).

### The cellular uptake of rhodamine-loaded NPs by SH-SY5Y cells

**Fig. 2:**
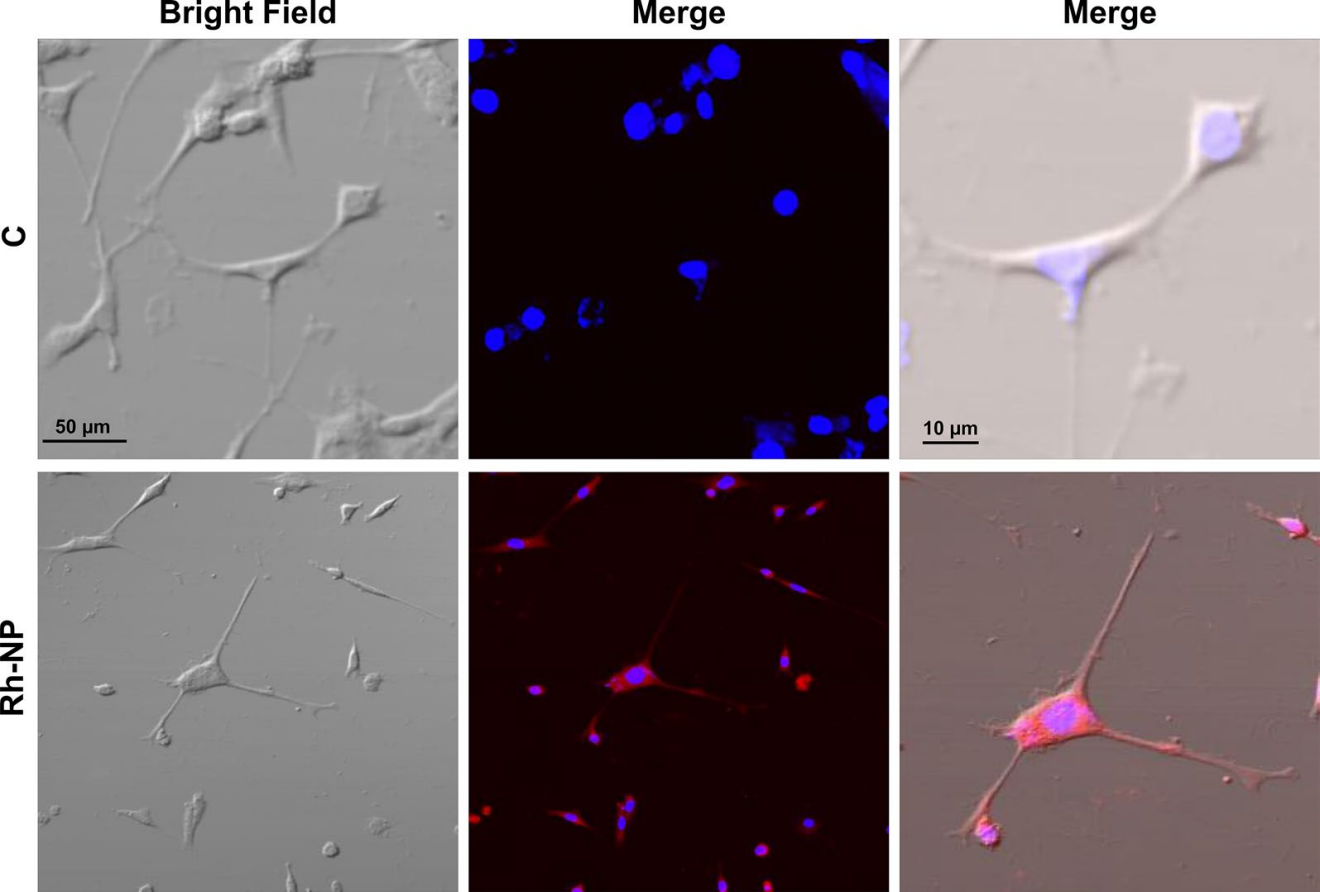
Microscopic study showing the cellular internalization of the rhodamin123-loaded PLGA@PEG NPs; Confocal laser scanning microscopy (CLSM) images of the SH-SY5Y cells treated with rhodamin123-loaded PLGA@PEG NPs for 12 h (*scale bar* 50 and 10 µm)

The cellular uptake analyses experiment was conducted to evaluate the ability of SH-SY5Y cells to engulf NPs. In order to investigate the intracellular retention, rhodamine-loaded NPs were supplemented in DMEM media to cultured cells incubated for 12 h. The microphotographs images shown in Fig. 2 reveal that rhodamine-loaded NPs were efficiently engulfed and internalized by SH-SY5Y cells.

### An-NPs prevented Aβ_1–42_-induced P38/JNK pathways activation in vitro

**Fig. 3:**
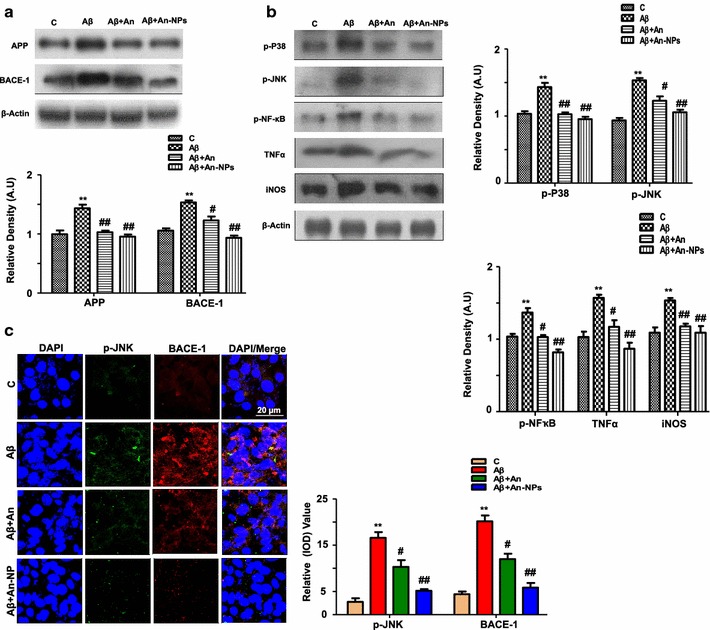
An-NPs attenuated Aβ pathology and prevents Aβ_1–42_-induced activation of P38/JNK Pathways. **a** Effect of An-NPs treatment on the expression of Alzheimer markers analyzed by Western Blot in Aβ_1–42_ treated SHSY-5Y cell line. Shown are representative Western Blots probed with antibodies of APP and BACE-1 in the Aβ_1–42_ treated SHSY-5Y cells. Data are the representative of three individual experiments (n = 3). The protein bands were quantified using sigma gel software. β-Actin was used to show equivalent amounts of protein loading. **b** Representative Western Blots of p-P38, p-JNK, p-NF-kB, TNF-α and iNOS in Aβ_1–42_-treated SH-SY5Y cells. Data are the representative of three individual experiments (n = 3). The protein bands were quantified using sigma gel software. β-Actin was used to show equivalent amounts of protein loading. **c** The double immunofluorescence images of SH-SY5Y cells after Aβ_1–42_ and An-NPs treatment, showing p-JNK (*green*) and BACE-1 (*red*), proteins and their respective relative density histograms. The DAPI (*blue*) was used to stain the nucleus. All the experiments were performed in triplicate. The details are given in the "Methods" section. *Significantly different from the control; ^#^significantly different from Aβ_1–42_-treated group. Significance = **p < 0.01, ^#^p < 0.05, ^##^p < 0.01

It is well established that Aβ deposition occurs in the development of AD [40]. Western blot analyses were performed to determine the proteins expression level of AD markers like APP and BACE-1 in the Aβ_1–42_ treated SH-SY5Y cells. The results showed that An-NPs treatment showed a significant reduction in the level of APP and BACE-1 compared to Aβ_1–42_ treated group (Fig. 3a).

To investigate the beneficial effect of An-NPs against Aβ_1–42_-induced activation of the P38/JNK pathway, we performed western blot. As the literature highlighted that the two important members of the MAPK family of proteins such as P38-MAPK and c-JNK mainly involved in stress [41, 42], and their activation has been implicated in oxidative stress and further triggers inflammatory mediators such as TNF-α, interleukins, and iNOS [43, 44]. The western blot results shown in Fig. 3b indicate that An-NPs treatment caused a significant reduction in p-P38 and p-JNK protein expression compared with the Aβ_1–42_-treated group. Moreover, An-NPs treatment significantly decreased the expression of BACE-1 and p-JNK compared with the Aβ_1–42_-treated group and alone native anthocyanin as evaluated morphologically (Fig. 3c).

### An-NPs inhibited Aβ_1–42_-activated NF-κB and various inflammatory protein markers expression in vitro

Activation of the MAPK family of proteins induces the phosphorylation of other protein kinases and the generation of cytotoxic factors and proinflammatory cytokines [42]. To evaluate the effect of An-NPs on different inflammatory mediators, western blotting was performed to monitor the expression levels of phosphorylated-NF-kB, TNF-α and iNOS proteins against Aβ_1–42_ in SH-SY5Y cell line. The results showed that Aβ_1–42_ supplementation significantly elevated the levels of these proteins in SH-SY5Y cells compared with the control cells. In contrast treatment of An-NPs significantly inhibited the Aβ_1–42_-induced increased expression of p-NF-kB, TNF-α and iNOS proteins (Fig. 3b).

### An-NPs treatment alleviated Aβ_1–42_-induced oxidative stress and upregulated the endogenous antioxidant system in vitro

**Fig. 4:**
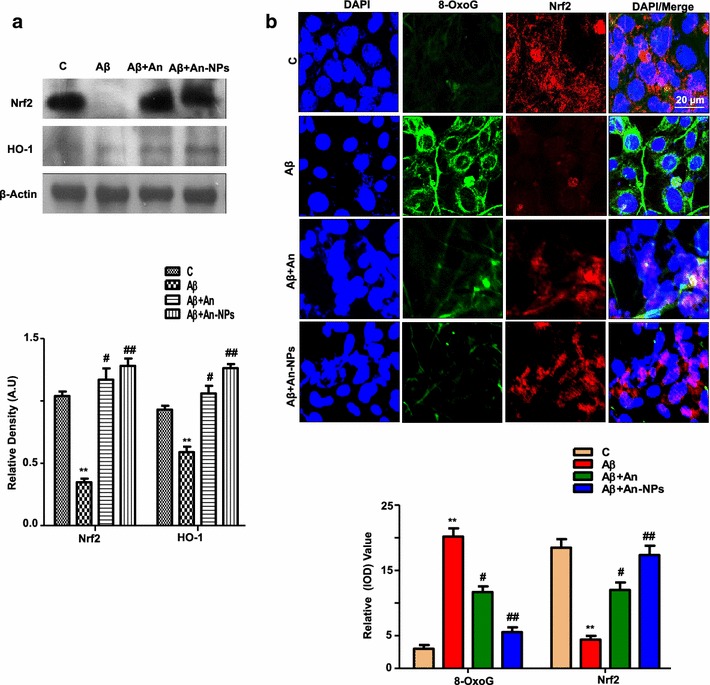
An-NPs treatment alleviated oxidative stress and upregulated Nrf-2 and its downstream targets genes HO-1 expressions. **a** Shown are representative Western Blots probed with antibodies of Nrf2, and HO-1 in the Aβ_1–42_ treated SHSY-5Y cells. Data are the representative of three individual experiments (n = 3). The protein bands were quantified using sigma gel software. β-Actin was used to show equivalent amounts of protein loading. **b** The double immunofluorescence images of SH-SY5Y cells after Aβ_1-42_ and An-NPs treatment, showing 8-Oxo-G (*green*) and Nrf2 (*red*), proteins and their respective relative density histograms. The DAPI (*blue*) was used to stain the nucleus. All the experiments were performed in triplicate. The details are given in the “Methods” section. *Significantly different from the control; ^#^significantly different from Aβ_1–42_-treated group. Significance = **p < 0.01, ^#^p < 0.05, ^##^p < 0.01

It has shown that Nrf-2 and its target anti-oxidant genes such as HO-1 has a critical role in the mechanism against oxidative stress and induced anti-oxidant mechanism that is assumed is beneficial in AD [45], while, Kanninen et al. report states that nuclear translocation of Nrf-2 increased the HO-1 expression [46]. The western blot results shown in Fig. 4a revealed that there are decreased levels of Nrf-2 and HO-1 proteins in the Aβ_1–42_-treated SH-SY5Y cells compared to the control group. On the other hand either anthocyanins alone (native) or in conjugation with nanoparticles (An-NPs) significantly upregulated the endogenous antioxidant genes such as Nrf-2 and HO-1 proteins expression against Aβ_1–42_ treated cells.

There are several studies which demonstrate that 8-oxoguanine (8-OxoG) is an oxidative stress marker and it is increased in the AD brain and APPswe/PS1deltaE9 transgenic AD mouse model [47]. Therefore, we analysed the colocalization of oxidative stress marker i.e. 8-OxoG and Nrf-2 through immunofluorescence staining. The immunofluorescence results showed increased immunoreactivity of 8-OxoG and lower Nrf-2 proteins expression in the Aβ_1–42_-treated SH-SY5Y cell line as compared to the untreated control cells. However, the cells received An-NPs has reduced immunoreactivity of 8-OxoG and increased the translocation of Nrf-2 proteins against Aβ_1–42_ in vitro (Fig. 4b). These results clearly indicate that An-NPs have more significant antioxidant activity compared to native anthocyanins (Fig. 4a, b).

### An-NPs prevented Aβ_1–42-_induced apoptosis and neurodegeneration in vitro

**Fig. 5:**
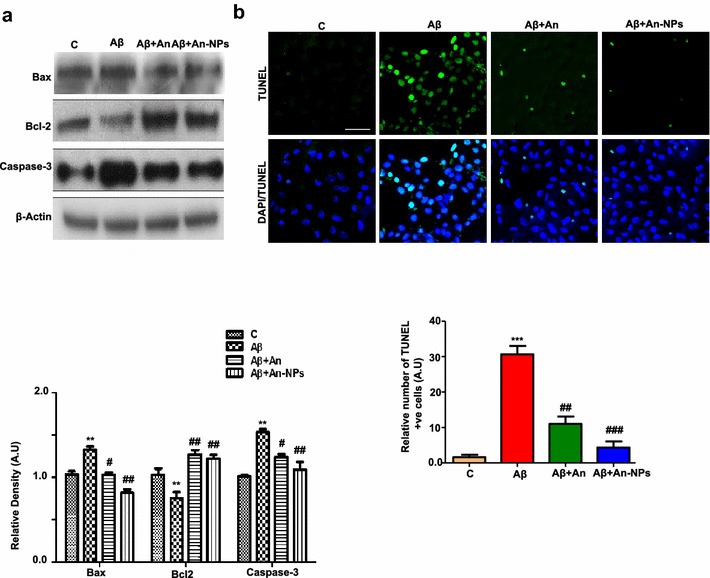
The beneficial effects of An-NPs against Aβ_1–42_-induced apoptosis and neurodegeneration; **a** effect of An-NPs treatment on the activation of apoptotic induced proteins analyzed by Western Blot in Aβ_1–42_ treated SH-SY5Y cell line. Induction of Bax, Bcl-2 and caspase-3 in Aβ_1–42_-treated SH-SY5Y cells were analyzed by Western Blot. Data are the representative of three individual experiments (n = 3). The protein bands were quantified using sigma gel software. β-Actin was used to show equivalent amounts of protein loading. **b** Representative photomicrographs of TUNEL and DAPI stained cells (magnification ×10 objective field, *scale bar* 50 µm) showing a high number of apoptotic neurons in Aβ_1–42_-treated group and comparatively low number of apoptotic neurons in Aβ plus An-NPs treated group. Data are the mean value (±SEM) for three independent experiments (n  =  3). *Significantly different from the control; ^#^significantly different from Aβ_1–42_-treated group. Significance = **p < 0.01, ***P < 0.001, ^#^p < 0.05, ^##^p < 0.01, ^###^p < 0.001

Previous published literature has determined the pro-apoptotic activity of Aβ_1–42_ and plays a critical role in neurodegeneration in AD [48]. The Bcl-2 family of proteins plays a crucial role in the intracellular apoptotic signal transduction. Mitochondrial apoptosis regulated by this family of proteins involves antiapoptotic proteins Bcl-2 and proapoptotic proteins Bax [49]. Our results showed that Aβ_1–42_ treatment significantly increase the expression of pro-apoptotic Bax proteins and decreases the expression of the anti-apoptotic proteins Bcl-2 compared to the control cells. Interestingly, the An-NPs significantly reversed the expression level of Aβ_1–42_-induced Bax and Bcl-2 proteins. Similarly, western blot analysis was performed to determine the protein expression level of caspase-3 following Aβ_1–42_ and An-NPs treatment. Our results showed that Aβ_1–42_ administration significantly increases the protein level of caspase-3 in the SH-SY5Y cells. Consistent with other results, An-NPs treatment along with Aβ_1–42_ showed a significant decrease in the level of caspase-3 (Fig. 5a). For morphological assessment of Aβ_1–42_-induced neuronal cell death, the antiapoptotic effects of An-NPs were examined using TUNEL staining in SH-SY5Y cells. The results showed that Aβ_1–42_ treatment induced DNA damage and increased the number of TUNEL positive cells compared with control group. Exposure to An-NPs following Aβ_1–42_ treatment significantly reduced the number of TUNEL positive cells in SH-SY5Y cell lines showing enhanced neuroprotection compared to alone free anthocyanin (Fig. 5b).

## Discussion

Nanotechnology is an emerging field that has been recognized to have the potential to make impacts on the detection, prevention and treatment of different human related diseases and disorders. For nearly last three decades, biodegradable PLGA nanoparticles have been extensively investigated as drug delivery systems for the treatment of several diseases [12]. In fact, nanoparticles can protect the encapsulated drug from degradation, release the drug in a controlled manner, improve its biodistribution and allow the drug to target the tissues [9]. In this study we developed anthocyanin loaded PLGA@PEG nanoparticles. The main advantage of using PLGA-NPs for drug delivery is their biodegradability, and therefore no surgery is needed to remove the implant after its function is no longer required [50, 51]. The aims of this work were to synthesize anthocyanins loaded biodegradable and biocompatible NPs for controlled delivery against Aβ_1–42_-induced intoxication and oxidative stress in SH-SY5Y cell line using PLGA biodegradable polymer as inner core and modifying the surface with nontoxic blood compatible material PEG to acts as outermost shell. Both PLGA and PEG are approved by the US FDA and European Medicine Agency (EMA) employed for various drug delivery systems in preclinical and in clinical studies [52, 53]. These nanoparticles were prepared and fully characterized in terms of their particle size, surface charge, encapsulation efficiency and in vitro anthocyanin release profile. Our prepared NPs were of the almost same size (in nanometer range), negative zeta potential and were suggested suitable for intracellular uptake as demonstrated by previously published reports [54]. A close examination of the TEM micrograph at higher magnification and the image with lower resolution showed spherically circular shaped NPs (Fig. 1a). The outer region was presumed to be the hydrophilic crystalline PEG segment, and the inner region was assigned to inner hydrophobic amorphous core [55]. The mean particle size of PLGA@PEG-anthocyanins nanoparticles was in the range of 120–165 nm, and the zeta potential around −12 mV, which is considered adequate to form stable dispersions. According to Lockman et al. [56], anionic charge on the nanoparticle supports entry through the blood–brain barrier when compared to the cationic nanoparticles. The repulsion among the high negatively charged NPs provides extra stability in aqueous solution [57]. Cell viability studies prove that An-NPs are not cytotoxic (Fig. 1c). The in vitro release of anthocyanins from An-NPs exhibits biphasic kinetics, which includes an initial burst release, caused by rapid drug diffusion from the surface of the nanospheres, followed by a sustained release that depends on drug diffusion and matrix erosion mechanisms. This biphasic profile was considered appropriate to obtain the desired effects. All the PEGylated nanoparticles showed similar release profiles of initial burst release of drug from the nanoparticles followed by a sustained release. The initial fast release of the drug from nanoparticles was suggested that some amount of the drug was absorbed on the surface or loosely bound to the inner polymer core and was lost during the initial stage of incubation while the strongly encapsulated drug in the core domains followed slow and sustained release kinetics [39].

The loading of the drugs in the form of nanocarriers makes an effective and site-specific delivery of the drug to its target site. Inside the cytoplasm, NPs remain longer and the drug is released slowly, resulting in a sustained therapeutic effect of the encapsulated agent [54]. The cellular uptake of rhodamine-loaded NPs was monitored using CLSM. The PEGylated NPs loaded with rhodamine was taken up by the SH-SY5Y cells and the red fluorescence which is characteristics of rhodamine was visualized clearly in the cytoplasm of the cells (Fig. 2). Some amount of fluorescence was also detected in the nucleus of the cell showing its intercalation in genomic DNA (Fig. 2). These results suggest that NPs were sufficiently internalized presumably via endocytosis like most of the other PEGylated drug conjugates [58]. The amorphous PEGylated NPs loaded with rhodamine was taken up by the prostate cancer DU145 cells [59].

Previous studies have shown that anthocyanins have antioxidant activities [60]. However, anthocyanins are unstable because their phenolic hydroxyl groups are easily oxidized into quinones, causing a reduced biological activity [61]. To overcome this drawback, anthocyanins are combined with macromolecules such as carbohydrates and proteins to increase their stability. Jiménez-Aguilar et al. reported that anthocyanins combined with polysaccharides, which can significantly prolong anthocyanin degradation time, have increased stability [62]. In the present study we showed that Aβ_1–42_ treatment induced oxidative stress by generating ROS in SH-SY5Y cell line. While, ROS activate various molecular signaling pathways. The free radical scavenging characteristics of An-NPs abrogated ROS production via alleviating the p38-MAPK/JNK signaling pathways. There can be multiple points in signaling pathways that mediate such pathway crosstalk when the components and their functional states of one pathway may affect the function of another pathway. In some cases, pathway crosstalk may be sustained by single proteins through molecular switches provided by post-translational modifications. Namely, different phosphorylation events may lead to inhibition or activation of the target protein and consequently potentially inhibit one pathway and activate another. These findings are in line with previous literature which demonstrates that both P38-MAPK and c-JNK (members of the MAPK family of proteins) mainly involved in stress conditions [41, 42]. Other studies also correlated the activation of these kinases with the induction of oxidative stress and stimulation of pro-inflammatory markers such as TNF-α, interleukins, and iNOS [43, 44]. In the current study we have conducted immunofluorescence analysis to know about the correlation between BACE1 and phosphorylated JNK after Aβ_1–42_ treatment. In contrast the An-NPs not only completely inhibited the activation and phosphorylation of JNK but also significantly reduced BACE1 expression in SH-SY5Y cells. Additionally, we have shown that An-NPs stimulated the activation of endogenous antioxidant genes such as Nrf2 and HO-1 to cope with the oxidative stress induced by Aβ_1–42_ in SH-SY5Y cell. The activation of the Nrf2/heme oxygenase-1 (HO-1) axis was responsible for the prosurvival effect against oxidative stress. Moreover, it has been investigated that activated p-JNK lead to neuroinflammation and neurodegeneration [31, 32]. Our current findings reveal that An-NPs scavenge ROS molecules and can also overcome the activation of kinases. In this regard we could say that these An-NPs evidenced the presence of a cross talk between different stress kinases and endogenous antioxidant genes by a sustained drug release characteristics. Interestingly, these An-NPs in comparative to free anthocyanins have shown more significant effects against Aβ_1–42_-induced oxidative stress, neuroinflammation and neurodegeneration in SH-SY5Y cell line.

The Aβ_1–42_ neurotoxicity is reported previously both in vitro and in vivo model [20, 21]. Oxidative stress is implicated in various neurodegenerative diseases including AD. ROS induced oxidative stress is considered to be a critical mediator in the AD pathology [22]. The anthocyanins have antioxidant properties with a free radical scavenging activity [60]. Similarly, here we found the elevated ROS and oxidative stress level and activated immunofluorescence reactivity of 8-OxoG in the Aβ_1–42_-treated SH-SY5Y cells. Interestingly, our natural anti-oxidant anthocyanins and An-NPs attenuated the elevated ROS level and oxidative stress in the Aβ_1–42_-treated SH-SY5Y cell lines.

Recently Zhang et al. found that activation of Nrf2/HO-1 signaling is protective against oxidative stress [63]. Numerous studies proposed that endogenous anti-oxidant genes such as Nrf-2 and HO-1 activation produced neuroprotection in AD [45]. Consistently in our Aβ_1–42_-treated SH-SY5Y cells the expression of Nrf-2 and HO-1 were decreased. Treatment with An-NPs activates the expression of Nrf-2 and HO-1 and comparatively An-NPs significantly increased the level of Nrf-2 and HO-1 as compared to the Aβ_1–42_-treated group.

Our results also assured that An-NPs were also more active and potent than alone anthocyanin as it significantly decreased the level of basic proteins associated with AD like APP and BACE-1 in Aβ_1–42_-treated SH-SY5Y cells. It has been determined that pharmacological inhibition of p38α-MAPK decreased the levels of the inflammatory cytokines TNF-α and IL-1β, and protected neuronal cells from synaptic protein loss and neurite degeneration [64]. Accordingly Wang et al. showed that JNK inhibition decreased the production of inflammatory mediators, and inhibited the apoptotic pathway [65]. Although the exact neuroprotective mechanism of anthocyanins during Aβ_1–42_ mediated activation of the P38 and JNK pathways is not clear, our results indicate that anthocyanins decreases the levels of p-P38 and p-JNK, which may decrease the production of inflammatory mediators such as p-NF-kB, TNF-α and iNOS. Hence we suggest that An-NPs are effective enough to behave as neuroprotective agent against Aβ_1–42-_induced neurotoxicity.

Studies have shown that p-JNK activation induces activator protein (AP)-1-dependent BAX and caspase activation, and as a result apoptosis will occur [32]. The result of western blot analysis showed that the induction, activation and cleavage of apoptotic markers were higher in An-NPs as compared to native anthocyanin. In addition, TUNEL and DAPI staining provided further supporting evidence for An-NPs uptake and predominant amount of drug accumulation in the nucleus of Aβ_1–42_-treated SH-SY5Y cells after internalization of NPs into the cytoplasm, which caused cellular DNA damage and the onset of apoptosis.

**Fig. 6:**
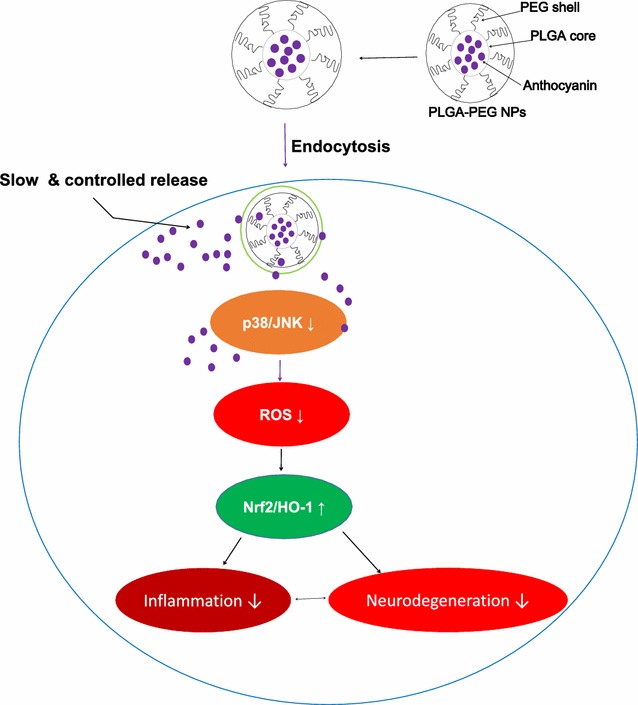
Schematic representation of intracellular uptake of anthocyanin loaded PEGylated NPs by SH-SY5Y cell via endocytosis; inside the cellular cytoplasm, the endosome is broken down by lysosomal enzymes and the drug is released in the cytoplasm and reverts the Aβ_1–42_-induced Aβ pathology by abrogating ROS generation via the P38-MAPK/JNK pathways accompanied by induction of endogenous nuclear factor erythroid 2-related factor 2 (Nrf2) and heme oxygenase 1 (HO-1)

Figure 6 showing the schematic representation of intracellular uptake of anthocyanin loaded PEGylated NPs by SH-SY5Y cell via endocytosis. Inside the cellular cytoplasm, the endosome is broken down by lysosomal enzymes and the drug is released in the cytoplasm and reverts the Aβ_1–42_-induced Aβ pathology by abrogating ROS generation via the P38-MAPK/JNK pathways accompanied by induction of endogenous nuclear factor erythroid 2-related factor 2 (Nrf2) and heme oxygenase 1 (HO-1).

## Conclusions

To improve the bioavailability of hydrophilic drugs PLGA@PEG nanoparticles were investigated as the drug delivery system. These NPs contributed to improving stability of encapsulated drugs against the outer environmental changes and to controlling drug release. Therefore, we assumed that PLGA@PEG nanoparticles have a potential as drug carriers for unstably hydrophilic drugs. Overall we demonstrate that that anthocyanin-loaded PLGA@PEG nanoparticles (An-NPs) formulation has enhanced cellular uptake and increased bioactivity in vitro and were not cytotoxic even at high doses. In summary, it was observed from the cellular study that An-NPs targeted Aβ_1–42_ treated SH-SY5Y cells more efficiently than native anthocyanins thereby alleviated Aβ pathology with reduction of ROS-induced oxidative stress through the activation of Nrf-2/HO-1 pathway, and consequently regulated the important neuronal P38-MAPK/JNK signalling and prevents the apoptosis and neurodegeneration through suppression of Bax, caspase-3 and TUNEL positive neuronal cells. Thus, it can be concluded that drug encapsulation in the PLGA@PEG nanocarriers could accumulate more drugs at the target site for sustained period of time relative to free drug in solution. In conclusion comparative to native anthocyanins, An-NPs effectively attenuated Aβ-induced neurotoxicity in SH-SY5Y cells and showed significant anti-oxidant, anti-apoptotic, anti-inflammatory and anti-Alzheimer’s effects and therefore we assume that An-NPs have more therapeutic potential to treat various neurological disorders like AD.

## Methods

### Materials

Poly (lactide-*co*-glycolide) (PLGA)–PEG, sodium deoxycholate, Aβ_1–42_ peptide and dichloromethane (DCM) were purchased from Sigma. All other chemicals used were of analytical grades. The anti-Bax, anti-Bcl-2, anti-APP, anti-BACE-1, anti-Nrf2, anti-HO-1, anti-p-NF-kB 65, anti-p-JNK, anti-TNFα, anti-iNOS, anti-p-P38, antibodies were purchased from Santa Cruz Biotechnology. Anti-caspase-3 and anti-actin antibodies were bought from Cell Signaling and anti-8-oxoguanine (anti-8-Oxo-G) were purchased from (Millipore, Billerica, MA, USA). The secondary antibodies used in our experiments were goat anti-mouse IgG, goat anti-rabbit IgG and rabbit anti-goat IgG, purchased from Santa Cruz Biotechnology.

### Preparation of anthocyanin loaded PLGA@PEG nanoparticles

Drug (Anthocyanin) loaded PLGA@PEG nanoparticles were prepared by way of a slightly modified emulsification-solvent evaporation technique [66]. Briefly, the PLGA@PEG 100 mg and drug 10 mg were dissolved in 3 ml of DCM under continuous stirring. This mixture was added to a 12 mM sodium deoxycholate solution (20 ml) and the mixture was probe sonicated at 20% power for 10 min using a microtip probe sonicator D-12207. The emulsion formed was then gently stirred in a fume hood at room temperature until complete evaporation of the organic phase achieved. The nanoparticles were purified by centrifugation at 12,000 rpm for 10 min and then washed with fresh water two times to remove the excessive emulsifier and untapped free drug. The nanoparticles suspension was freeze-dried in order to obtain a fine powder of the drug loaded nanoparticles. After centrifugation, the amount of anthocyanin in the supernatant was assayed by spectrophotometer (Hewlett Packard UV 8452A) at a wavelength of 520 nm. The drug encapsulation efficiency of PLGA-PEG nanoparticles was carried out as described previously [67]. Drug free nanoparticles were also prepared with the same method using single emulsion technique. For fluorescence-labeled NPs formulation, rhodamine-123 (0.5 mg/ml) was added to the inner aqueous phase and NPs were prepared using single emulsion–solvent evaporation method.

### Encapsulation efficiency

The encapsulation efficiency of anthocyanin was determined by the spectrophotometric method. The entrapment efficiency of anthocyanin in nanoparticles was found to be 60%. Briefly, 10 mg of anthocyanin nanoparticles was dissolved in 2 ml of DCM and kept in an incubator shaker with stirring for 30 min. The absorbance of the solution was measured at 520 nm. The amount of encapsulated anthocyanin was calculated from the standard curve drawn between varied amount of anthocyanin and absorbance, and all the measurements were carried out in triplicate. The encapsulation efficiency (EE) was determined from the following formula.

$$\begin{aligned}{\text{EE}}\,\% \,  &= \,{\text{weight of anthocyanin in nanoparticles}}/ \\  & \quad \; \,{\text{weight of anthocyanin used for nanoparticle preparation }} \\ & \quad \times \, 100 \end{aligned}$$

### Physiochemical properties of nanoparticles

The nanoparticle surface morphology was examined using TEM (transmission electron microscopy) (Tecnai-12, 120 kV). A small quantity of aqueous solution of the lyophilized anthocyanin-loaded nanoparticles (1 mg/ml) was placed on a TEM grid surface with a filter paper (Whatman No. 1). One drop of 10% uranyl acetate was added to the surface of the carbon-coated grid. After 1 min of incubation, excess fluid was removed and the grid surface was air dried at room temperature. It was then loaded into the transmission electron microscope attached to a Gatan SC 1000 CCD camera.

The zeta-potential and mean particle size of anthocyanin-loaded nanoparticles were measured by dynamic laser light scattering using a (ELS-Z, DLS-8000; Otsuka Electronics Co., Osaka, Japan). The nanoparticles were suspended in water at a concentration of 1 mg/ml. The mean particles’ size and charge were measured at 25 ± 2 °C, by following settings in the Zetasizer: nominal 5 mW He–Ne laser operating at 633 nm wavelength; viscosity for water 0.89 cP, and refractive index of water 1.33. Zeta-potential values were presented as an average value of 30 runs, with triplicate measurements within each run. The mean particle size of the NPs was determined in triplicate, and the average values were calculated.

Fourier transform-infra red (FT-IR) spectra were observed to study the interaction of anthocyanin with NPs under vacuum on a VERTEX 80v (Bruker Optics) FT-IR spectrometer equipped with a DTGS (with KBr window) detector. Freeze-dried samples were mixed with KBr powder, ground at room temperature and by an agate mortar to be finally compressed into a thin tablet. The study was done in triplicate with the scanning range set between 400 and 4000 cm^−1^.

### In vitro drug release

The measurement of anthocyanin release from the drug loaded nanoparticles in vitro was carried out in a glass apparatus containing 50 ml of PBS (pH 7.4) at 37 °C as described previously [68]. In brief, 50 mg of the drug loaded nanoparticles was dispersed in 5 ml of PBS and placed into a cellulose membrane dialysis tube (molecular weight cutoff = 3000–3500 Da). The dialysis tube was then immersed in the release medium (50 ml) and incubated in a shaker bath (100 rpm) at 37 °C. Aliquots of 1 ml were periodically withdrawn from the solution. The volume of the solution was held constant by adding 1 ml of fresh buffer solution after each sampling to ensure sink condition. The amount of anthocyanin released in the medium was analyzed spectrometrically at 520 nm. The percent release of the anthocyanin was then plotted as a function of dialysis time.

For the control experiment, 5 mg of free anthocyanin was dissolved in DCM and poured into 5 ml of PBS and sonicated. The organic solvent was evaporated by stirring and most anthocyanin remained dissolved in the medium with little amount of suspended anthocyanin particles. All experiments were repeated in triplicate.

### Cell culture and drug treatment

Amyloid-β_1–42_ (Sigma-Aldrich, St. Louis, MO, USA) was dissolved in sterile saline at concentration of 1 mg/ml to prepare stock solution. This solution was incubated at 37 °C for 4 days. The SH-SY5Y cells (purchased from Korean Cell Bank, South Korea) were maintained in a solution DMEM, 10% fetal bovine serum (FBS) and antibiotics (penicillin and streptomycin), grown for 5 days and treated as follows: (1) Control: incubated in DMEM solution for 24 h; (2) Aβ_1–42_-treatment: incubated in DMEM solution containing Aβ_1–42_ (5 µM) for 24 h; (3) Aβ_1–42_ + native anthocyanin treatment: incubated in DMEM solution containing Aβ_1–42_ (5 µM) for 12 h and then post incubated with native anthocyanin (200 µg/ml) for 12 h; (4) Aβ_1–42_ + An-NPs treatment: incubated in DMEM solution containing Aβ_1–42_ (5 µM) for 12 h and then post incubated with An-NPs (200 µg/ml) for 12 h; All the cells were harvested at day 6 and used for the desired analysis.

### MTT assay

First we checked the in vitro cytotoxicity of PLGA@PEG nanoparticles (without anthocyanin), native anthocyanin and anthocyanin loaded PLGA@PEG nanoparticles (An-NPs) in normal SH-SY5Y cells by 3-(4,5-dimethylthiazol-2-yl)-2,5-diphenyltetrazolium bromide (MTT) assay. Four different concentrations of each sample were tested in normal SH-SY5Y cell line. The cells were seeded in 96-well plates with 1 × 10^4^ cells/well and incubated with increasing concentrations of equivalent anthocyanin ranging from 50 to 200 μg/ml incubated at 37 °C for 24 h. The cells treated with the growth medium served as control. Following this, the cells were incubated with MTT solution for another 2–4 h at 37 °C. Subsequently, the medium in each well was replaced with DMSO. Finally, the absorbance of the solution in each well at 570 nm was measured using an ApoTox (Promega) instrument. All experiments were performed independently in triplicate. Further we confirmed the viability of the SH-SY5Y against Aβ_1–42_ and was assessed by performing the MTT assay according to the manufacturer’s instructions (Sigma Aldrich). Briefly, the cells were cultured in 96-well plates at a density of 1 × 10^4^ cells per well in 100 µl of the Dulbecco’s modified Eagle’s medium (DMEM from Gibco, life technologies, USA). After 24 h, the medium was replaced with fresh medium containing Aβ_1–42_ (5 µM), with three different concentrations (50, 100 and 200 µg/ml) of anthocyanin or An-NPs in combination with Aβ_1–42_ (5 µM). The control cells received only the DMEM medium. The cytotoxicity of the cells was measured as described previously [69].

### Oxidative stress (ROS) detection in vitro

The ROS assay in SH-SY5Y cells were conducted as described previously [70]. Briefly, the cells were cultured in 96-well plates at a density of 1 × 10^4^ cells per well in 100 µl of the Dulbecco’s modified Eagle’s medium (DMEM from Gibco, life technologies, USA). After 24 h, the medium was replaced with fresh medium containing Aβ_1–42_ (5 µM), with three different concentrations (50, 100 and 200 µg/ml) of anthocyanin or An-NPs in combination with Aβ_1–42_ (5 µM). The control cells received only the DMEM medium. Following this, A 600-µM solution of DCFDA (20, 70-dichlorofluorescein diacetate) dissolved in DMSO/PBS was then added to each well, and the cells were incubated for 30 min. The plates were then read on an ApoTox-Glo (Promega) instrument at 488/530 nm.

### ApoTox-Glo triplex assay

The ApoTox-Glo Triplex Assay (Promega) was performed to assess viability, cytotoxicity and caspase-3/7 activation within a single assay well. The assay consists of two parts: in the first part, the activities of two proteases, which are markers of cell viability and cytotoxicity, were measured simultaneously. SHSY-5Y cells (2 × 10^4^ cells) were cultured in 96-well assay plates. Each well contained a final volume of 200 µl of DMEM containing 10% FBS and 1% penicillin/streptomycin. After 48 h of incubation at 37 °C in a humidified 5% CO_2_ incubator, the cells were treated with Aβ_1–42_, native anthocyanin and anthocyanin loaded PLGA@PEG nanoparticles as explained in the drug treatment section. For the assay, 20 µl of the viability/cytotoxicity reagent containing both GF-AFC substrate and bis-AAF-R110 substrate was added to all the wells, briefly mixed via orbital shaking (500 rpm for 30 s) and incubated for 1 h at 37 °C. The fluorescence was measured at two wavelengths: 400/505 nm (viability) and 485/520 nm (cytotoxicity). The GF-AFC substrate enters live cells and is cleaved by a live-cell protease to release AFC. The bis-AAF-R110 substrate does not enter live cells but rather is cleaved by a dead-cell protease to release R110. The live-cell protease activity is restricted to intact viable cells and is measured using a fluorogenic, cell-permeant peptide substrate [glycyl-phenylalanyl-aminofluo rocoumarin (GFAFC)]. A second fluorogenic, cell-impermeant peptide substrate [bis-alanylalanylphenylalanyl-rhodamine 110 (bis-AAF-R110)] was used to measure the activity of dead-cell proteases that are released from cells that have lost membrane integrity. The second part of the assay uses a luminogenic caspase-3 substrate, containing the tetrapeptide sequence DEVD, to measure caspase activity, luciferase activity and cell lysis. The caspase-Glo3/7 reagent was added (100 ml) to all the wells and briefly mixed using orbital shaking (500 rpm for 30 s). After incubation for 30 min at room temperature, the luminescence was measured to determine caspase activation.

### Cellular uptake

The uptake of rhodamine-loaded PLGA@PEG NPs was studied in normal SH-SY5Y cell line using confocal laser scanning microscopy (CLSM). For CLSM (Fluoview FV 1000, Olympus, Japan) observation, freshly prepared rhodamine-123-loaded NPs suspension at concentration of 0.1 mg/ml was mixed with cell culture medium and added to the cells pre-cultured in 4-well cover glass chambers and incubated for 12 h. However, to the control group cells (cells not treated with rhodamine-loaded PLGA@PEG NPs), only the culture medium was changed and applied the same condition. Following incubation, the cells were washed three times with PBS and were fixed with 4% paraformaldehyde and again washed with PBS and incubated with DAPI for 5 min. The slides were then rinsed with PBS and glass cover slips were mounted on glass slides with mounting medium, and fluorescent images were captured with CLSM.

### Western blot analysis

The western blot analysis were conducted as described previously [71]. Cells were harvested and then homogenized in 0.2 M PBS containing protease inhibitor cocktail. After centrifugation the respective protein samples were collected from each group and run through SDS-PAGE on 7–18% gels under reducing conditions. GangNam-STAIN (iNtRON Biotechnology) protein marker; 7–200 kDa was run in parallel for detection of the molecular weights of the proteins. The proteins were then transferred onto a polyvinylidene difluoride (PVDF) membrane and blocked in 5% skimmed milk. Immunoblotting were performed with respective primary antibodies. Anti-actin antibody served as loading control. The membranes were then probed with a goat derived horseradish peroxidase-conjugated anti-rabbit IgG or anti-goat IgG or anti-mouse IgG secondary antibodies (Santa Cruz Biotechnology, CA, USA). The immunoreactions over the PVDF membrane were visualized using Ez West Lumi western blotting detection reagent (ATTO Corporation, Japan). The x-ray films were scanned and analyzed with computer based Sigma Gel software (Jandel Scientific, San Rafael, and Chicago, USA) to get the resultant band densities.

### TUNEL assay

Terminal deoxynucleotidyl transferase (TdT)-mediated dUTP nick end labeling (TUNEL) staining was performed according to the manufacturer recommendations to determine apoptotic cell death. In situ cell death detection kit was purchased from Roche (Cat. No. 11684809910). Confluent SH-SY5Y cell lines were treated as described in drug treatment section with blank media, Aβ_1–42_-treatment, Aβ_1–42_ + anthocyanin and Aβ_1–42_ + An-NPs. To observe typical features of apoptosis, nuclear DNA was stained with terminal deoxynucleotidyl transferase (TdT)-mediated dUTP nick end-labeling (TUNEL) for 45 min (GenScript Corporation, USA). The cells were washed twice with PBS, and then counterstained with 4,6-diamidino-2-phenylindole (DAPI) for 10 min (Molecular Probes, Eugene, OR, USA). Glass cover slips were mounted on glass slides with mounting medium. TUNEL-positive (green) and DAPI (blue) staining patterns were acquired by use of a confocal laser scanning microscope (Fluoview FV 1000, Olympus, Japan).

### Immunofluorescence staining

Briefly, the slides containing SH-SY5Y cells were washed twice for 10 min each in 0.01 M PBS and incubated for 1 h in blocking solution containing 2% normal bovine serum (Santa Cruz Biotechnology), according to the antibody treatment, and 0.3% Triton X-100 in PBS. After blocking, the slides were incubated overnight at 4 °C with anti-p-JNK, anti-BACE-1, and anti-Nrf2, (Santa Cruz Biotechnology) and mouse monoclonal anti-8-Oxo-G (Millipore) antibodies diluted 1:100 in blocking solution. Following this, the slides were incubated for 2 h with the fluorescein isothiocyanate FITC/TRITC-labeled secondary antibodies (1:50) (Santa Cruz Biotechnology). The slides were then counterstained with 40,6-diamidino-2-phenylindole (DAPI) for 10 min and mounted with the Prolong Anti-fade Reagent (Molecular Probe, Eugene, OR, USA). Staining images of the double immunofluorescence were examined using a confocal laser-scanning microscope (Flouview FV 1000, Olympus, Japan).

### Data analysis

The Western blot bands were scanned and analyzed through densitometry using the Sigma Gel System (SPSS Inc., Chicago, IL). One-way analysis of variance (ANOVA) followed by a two-tailed independent Student’s *t* test were used for comparisons among the treated groups and the control. The Image-J software was used for immunohistological quantitative analysis. The density values of the data were expressed as the means ± SEM of three independent experiments. p values less than 0.05 were considered to be statistically significant. *p < 0.05, **p < 0.01 and ***p < 0.001; and ^**#**^p < 0.05, ^##^p < 0.01 and ^###^p < 0.001. *Significantly different from the control group; ^#^significantly different from the Aβ_1–42_-treated group.

## Additional file

**Additional file 1.** Additional figures.

## Abbreviations

PLGA: poly (lactide-*co*-glycolide)
PEG: polyethylene glycol
TEM: transmission electron microscopy
DLS: dynamic light scattering
FT-IR: Fourier transform infrared spectroscopy
ROS: reactive oxygen species
8-Oxo-G: 7, 8-dihydro-8-oxoguanine
Nrf2: nuclear factor erythroid 2-related factor 2
HO-1: heme oxygenase 1
p-JNK: phospho-c Jun N terminal kinase
p-NF-*k*B: phospho-nuclear factor kappa B
TNF-α: tumor necrosis Factor-α
iNOS: inducible nitric oxide synthase
APP: amyloid precursor protein
BACE-1: beta-site amyloid precursor protein cleaving enzyme 1
TUNEL: terminal deoxnucleotidyl transferase dUTP nick end labeling
FITC: fluorescein isothiocyanate
DAPI: 4′,6-diamidino-2-phenylindole
MTT: 3-4,5-dimethylthiazol-2-Yl-2,5-diphenyltetrazolium bromide
DMEM: Dulbecco’s modified Eagle’s medium

## References

[1] Torchilin VP, Lukyanov AN (2003). Peptide and protein drug delivery to and into tumors: challenges and solutions. Drug Discov Today.

[2] Orton CG (1995). Width of the therapeutic window: what is the optimal dose-per-fraction for high dose rate cervix cancer brachytherapy?. Int J Radiat Oncol Biol Phys.

[3] Sahoo SK, Dilnawaz F, Krishnakumar S (2008). Nanotechnology in ocular drug delivery. Drug Discov Today.

[4] Bangham AD (1993). Liposomes: the Babraham connection. Chem Phys Lipids.

[5] Chiappetta DA, Sosnik A (2007). Poly (ethylene oxide)-poly (propylene oxide) block copolymer micelles as drug delivery agents: improved hydrosolubility, stability and bioavailability of drugs. Eur J Pharm Biopharm.

[6] Labhasetwar V, Song C, Levy RJ (1997). Nanoparticle drug delivery system for restenosis. Adv Drug Deliv Rev.

[7] Westesen K, Bunjes H, Koch MHJ (1997). Physicochemical characterization of lipid nanoparticles and evaluation of their drug loading capacity and sustained release potential. J Control Release.

[8] Singh R, Lillard JJ (2009). Nanoparticle-based targeted drug delivery. Exp Mol Pathol.

[9] Kumari A, Yadav SK, Yadav SC (2010). Biodegradable polymeric nanoparticles based drug delivery systems. Colloids Surf B Biointerfaces.

[10] Prokop A, Davidson JM (2008). Nanovehicular intracellular delivery systems. J Pharm Sci.

[11] Vert M, Mauduit J, Li S (1994). Biodegradation of PLA/GA polymers: increasing complexity. Biomaterials.

[12] Danhier F, Ansorena E, Silva JM, Coco RL, Breton A, Préat V (2012). PLGA-based nanoparticles: an overview of biomedical applications. J Control Release.

[13] Mosqueira VC, Legrand P, Gulik A, Bourdon O, Gref R, Labarre D, Barratt G (2001). Relationship between complement activation, cellular uptake and surface physicochemical aspects of novel PEG-modified nanocapsules. Biomaterials.

[14] Duan Y, Sun X, Gong T, Wang Q, Zhang Z (2006). Preparation of DHAQ-loaded mPEG–PLGA–mPEG nanoparticles and evaluation of drug release behaviors in vitro/in vivo. J Mater Sci Mater Med.

[15] Gref R, Minamitake Y, Peracchia MT, Trubetskoy V, Torchilin V, Langer R (1994). Biodegradable long-circulating polymeric nanospheres. Science.

[16] Avgoustakis K, Beletsi A, Panagi Z, Klepetsanis P, Livaniou E, Evangelatos G, Ithakissios DS (2003). Effect of copolymer composition on the physicochemical characteristics, in vitro stability, and biodegradation of PLGA–mPEG nanoparticles. Int J Pharm.

[17] Kumar V, Prud’homme RK (2008). Thermodynamic limits on drug loading in nanoparticle cores. J Pharm Sci.

[18] Basu S, Harfouche R, Soni S, Chimote G, Mashelkar RA, Sengupta S (2009). Nanoparticles-mediated targeting of MAPK signaling predisposes tumor to chemotherapy. PNAS.

[19] Coomaraswamy J, Kilger E, Wölfing H, Schäfer C, Kaeser SA, Wegenast-Braun BM, Hefendehl JK, Wolburg H, Mazzella M, Ghiso J, Goedert M, Akiyama H, Garcia-Sierra F, Wolfer DP, Mathews PM, Jucker M (2010). Modeling familial Danish dementia in mice supports the concept of the amyloid hypothesis of Alzheimer’s disease. Proc Natl Acad Sci USA.

[20] Chan KH, Lam KSL, Cheng OY (2012). Adiponectin is protective against oxidative stress induced cytotoxicity in amyloid-beta neurotoxicity. Plos ONE.

[21] Lamert MP, Barlow AK, Chromy BA (1998). Diffusable, nonfibrillar ligands derived from Aβ_1–42_ are potent central nervous system neurotoxins. Proc Natl Acad Sci USA.

[22] Butterfield DA (1997). beta-Amyloid-associated free radical oxidative stress and neurotoxicity: implications for Alzheimer’s disease. Chem Res Toxicol.

[23] Von-Otter M (2010). Nrf2- encoding NFE2L2 haplotypes influence disease progression but not risk in Alzheimer’s disease and age-related cataract. Mech Ageing Dev.

[24] Barone E (2012). Heme oxygenase-1posttranslational modifications in the brain of subjects with Alzheimer disease and mild cognitive impairment. Free Radic Biol Med.

[25] Zou Y (2013). Protective effect of puerarin against beta-amyloid induced oxidative stress in neuronal cultures from rat hippocampus: involvement of the GSK-3β/Nrf2 signaling pathway. Free Radic Res.

[26] Kanninen K (2008). Nuclear factor erythroid 2-related factor 2 protects against beta amyloid. Mol Cell Neurosci.

[27] Shoji M, Iwakami N, Takeuchi S, Waragai M, Suzuki M, Kanazawa I, Lippa CF, Ono S, Okazawa H (2000). JNK activation is associated with intracellular β-amyloid accumulation. Brain Res Mol Brain Res.

[28] Zhu X, Raina AK, Rottkamp CA, Aliev G, Perry G, Boux H, Smith MA (2001). Activation and redistribution of c-Jun N-terminal kinase/stress activated protein kinase in degenerating neurons in Alzheimer’s disease. J Neurochem.

[29] Hensley K, Floyd RA, Zheng NY, Nael R, Robinson KA, Nguyen X, Pye QN, Stewart CA, Geddes J, Markesberry WR, Patel E, Johnson GVW, Bing G (1999). p38 kinase is activated in Alzheimer’s disease brain. J Neurochem.

[30] McDonald DR, Bamberger ME, Combs CK, Landreth GE (1998). β-Amyloid fibrils activate parallel mitogen-activated protein kinase pathways in microglia and THP1 monocytes. J Neurosci.

[31] Stambe C, Atkins RC, Hill PA, Nikolic-Paterson DJ (2003). Activation and cellular localization of the p38 and JNKMAPK pathways in rat crescentic glomerulonephritis. Kidney Int.

[32] Putcha GV, Le S, Frank S, Besirli CG, Clark K, Chu B, Alix S, Youle RJ, LaMarche A, Maroney AC, Johnson EMJ (2003). JNK-mediated BIM phosphorylation potentiates BAX-dependent apoptosis. Neuron.

[33] Shih PH, Yeh CT, Yen GC (2007). Anthocyanins induce the activation of phase 11 enzymes through the antioxidant response element pathway against oxidative stress-induced apoptosis. J Agric Food Chem.

[34] Joseph JA, Denisova NA, Arendash G, Gordon M, Diamond D, Shukitt-Hale B, Morgan D (2003). Blueberry supplementation enhances signaling and prevents behavioral deficits in an Alzheimer’s disease model. Nutr Neurosci.

[35] Zafra-Stone S, Yasmin T, Bagchi M, Chatterjee A, Vinson JA, Bagchi D (2007). Berry anthocyanins as novel antioxidants in human health and disease prevention. Mol Nutr Food Res.

[36] Bagchi D, Sen CK, Bagchi M, Atalay M (2004). Anti-antigenic, antioxidant, and anti-carcinogenic properties of a novel anthocyanin-rich berry extract formula. Biochemistry (Mosc).

[37] Hou DX (2003). Potential mechanisms of cancer chemoprevention by anthocyanins. Curr Mol Med.

[38] Varadinova MG, Docheva-Drenska DI, Boyadjieva NI (2009). Effects of anthocyanins on learning and memory of ovariectomized rats. Menopause.

[39] Allen C, Maysinger D, Eisenberg A (1999). Nano-engineering block copolymer aggregates for drug delivery. Colloids Surf B Biointerfaces.

[40] Selkoe DJ (2000). The origins of Alzheimer’s disease: a is for amyloid. JAMA.

[41] Zarubin T, Han J (2005). Activation and signaling of p38 MAP kinase pathway. Cell Res.

[42] Kyriakis JM, Ayruch J (2012). Mammalian MAPK signal transduction pathways activated by stress and inflammation: a 10-year update. Physiol Rev.

[43] Ajizan SJ, English BK, Meals EA (1999). Specific inhibitors of p38 and extracellular signal-regulated kinase mitogen-activated protein kinase pathways block inducible nitric oxide synthase and tumor necrosis factor accumulation in murine macrophages stimulated with lipopolysaccharide and interferon-gamma. J Infect Dis.

[44] Han Z, Boyle DL, Chang L, Bennett B, Karin M, Yang L, Manning AM, Firestein GS (2001). C-Jun N-terminal kinase is required for metalloproteinase expression and joint destruction in inflammatory arthritis. Clin Investig.

[45] Li L, Li W, Jung SW, Lee YW, Kim YH (2011). Protective effects of decursin and decursinol angelate against amyloid β-protein-induced oxidative stress in the PC12 cell line: the role of Nrf2 and antioxidant enzymes. Biosci Biotechnol Biochem.

[46] Kanninen K (2009). Intrahippocampal injection of a lentiviral vector expressing Nrf2 improves spatial learning in a mouse model of Alzheimer’s disease. Proc Natl Acad Sci USA.

[47] Hamilton A, Holscher C (2012). The effect of ageing on neurogenesis and oxidative stress in the APPswe/PS1deltaE9 mouse model of Alzheimer’s disease. Brain Res.

[48] Cancino LG (2008). STI571 prevents apoptosis, tau phosphorylation and behavioral impairments induced by Alzheimer’s β -amyloid deposits. Brain.

[49] Chao DT, Korsmeyer SJ (1998). BCL-2 family: regulators of cell death. Annu Rev Immunol.

[50] Yamamoto H, Tahara K, Kawashima Y (2012). Nanomedical system for nucleic acid drugs created with the biodegradable nanoparticle platform. J Microencapsul.

[51] Pridgen EM, Langer R, Farokhzad OC (2007). Biodegradable, polymeric nanoparticle delivery systems for cancer therapy. Nanomedicine (Lond).

[52] Fishburn CS (2008). The pharmacology of PEGylation: balancing PD with PK to generate novel therapeutics. J Pharm Sci.

[53] Tang Y, Sing J (2009). Biodegradable and biocompatible thermosensitive polymers based injectable implant for controlled release of protein. Int J Pharm.

[54] Panyam J, Labhasetwar V (2004). Sustained cytoplasmic delivery of drugs with intracellular receptors using biodegradable nanoparticles. Mol Pharm.

[55] Li X, Li R, Qian X, Ding Y, Tu Y, Guo R, Hub Y, Jiang X, Guo W, Liu B (2008). Superior antitumor efficiency of cisplatin-loaded nanoparticles by intratumoral delivery with decreased tumor metabolism rate. Eur J Pharm Biopharm.

[56] Lockman PR, Koziara JM, Mumper RJ, Allen DD (2004). Nanoparticle surface charges alter blood-brain barrier integrity and permeability. J Drug Target.

[57] Liu Y, Li K, Liu B, Feng SS (2010). A strategy for precision engineering of nanoparticles of biodegradable copolymers for quantitative control of targeted drug delivery. Biomaterials.

[58] Bareford LM, Swaan PW (2007). Endocytic mechanisms for targeted drug delivery. Adv Drug Del Rev.

[59] Shah M, Ullah N, Choi MH, Kim MO, Yoon SC (2012). Amorphous amphiphilic P(3HV-co-4HB)-b-mPEG block copolymer synthesized from bacterial copolyester via melt transesterification: nanoparticle preparation, cisplatin-loading for cancer therapy and in vitro evaluation. Eur J Pharm Biopharm.

[60] Wang BC, He R, Li ZM (2010). The stability and antioxidant activity of anthocyanins from blueberry. Food Technol Biotech.

[61] Giusti MM, Wrolstad RE (2003). Acylated anthocyanins from edible sources and their applications in food systems. Biochem Eng J.

[62] Jiménez-Aguilar DM, Ortega-Regules AE, Lozada-Ramírez JD, Pérez-Pérezd MCI, Vernon-Cartere EJ, Welti-Chanesa J (2011). Color and chemical stability of spray-dried blueberry extract using mesquite gum as wall material. J Food Compos Anal.

[63] Zhang H, Liu YY, Jiang Q, Li K, Zhao Y (2014). Salvianolic acid A protects RPE cells against oxidative stress through activation of Nrf2/HO-1 signaling. Free Radic Biol Med.

[64] Munoz L, Ranaivo HR, Rov SR, Hu W, Craft JM, McNamara LK, Chico LW, Van Eldik LJ, Watterso DM (2007). A novel P38MAPK inhibit or suppresses brain proinflammatory cytokine. J Neuroinflammation.

[65] Wang LW, Tu YF, Huang CC, Ho CJ (2012). JNK signaling is the shared pathway linking neuroinflammation, blood–brain barrier disruption, and oligodendroglial apoptosis in the white matter injury of the immature brain. J Neuroinflammation.

[66] Budhian A, Siegel SJ, Winey KI (2007). Haloperidol-loaded PLGA nanoparticles: systematic study of particle size and drug content. Int J Pharm.

[67] Tiwari SK, Agarwal S, Seth B, Yadav A, Nair S, Bhatnagar P, Karmakar M, Kumari M, Chauhan LK, Patel DK, Srivastava V, Singh D, Gupta SK, Tripathi A, Chaturvedi RK, Gupta KC (2014). Curcumin-loaded nanoparticles potently induce adult neurogenesis and reverse cognitive deficits in Alzheimer’s disease model via canonical Wnt/β-catenin pathway. ACS Nano.

[68] Prabaharan M, Grailer JJ, Pilla S, Steeber DA, Gong S (2009). Amphiphilic multi-arm block copolymer based on hyperbranched polyester, poly(l-lactide) and poly(ethylene glycol) as a drug delivery carrier. Macromol Biosci.

[69] Shah M, Naseer MI, Choi MH, Kim MO, Yoon SC (2010). Amphiphlic PHA-mPEG copolymeric nanocontainers for drug delivery: preparation characterization and in vitro evaluation. Int J Pharm.

[70] Amin FU, Shah SA, Kim MO (2016). Glycine inhibits ethanol-induced oxidative stress, neuroinflammation and apoptotic neurodegeneration in postnatal rat brain. Neurochem Int.

[71] Shah SA, Yoon GH, Chung SS, Abid MN, Kim TH, Lee HY, Kim MO (2016). Novel osmotin inhibits SREBP2 via the AdipoR1/AMPK/SIRT1 pathway to improve Alzheimer’s disease neuropathological deficits. Mol Psychiatry.

