# Supplementary material for: Anthocyanins encapsulated by PLGA@PEG nanoparticles potentially improved its free radical scavenging capabilities via p38/JNK pathway against Aβ1–42-induced oxidative stress
Source: J Nanobiotechnology. 2017 Feb 7;15:12. doi: 10.1186/s12951-016-0227-4 (PMC5297201; doi:10.1186/s12951-016-0227-4)
Supplement: Supplementary file 1 — Additional file 1. Additional figures. [file 12951_2016_227_MOESM1_ESM.pdf]

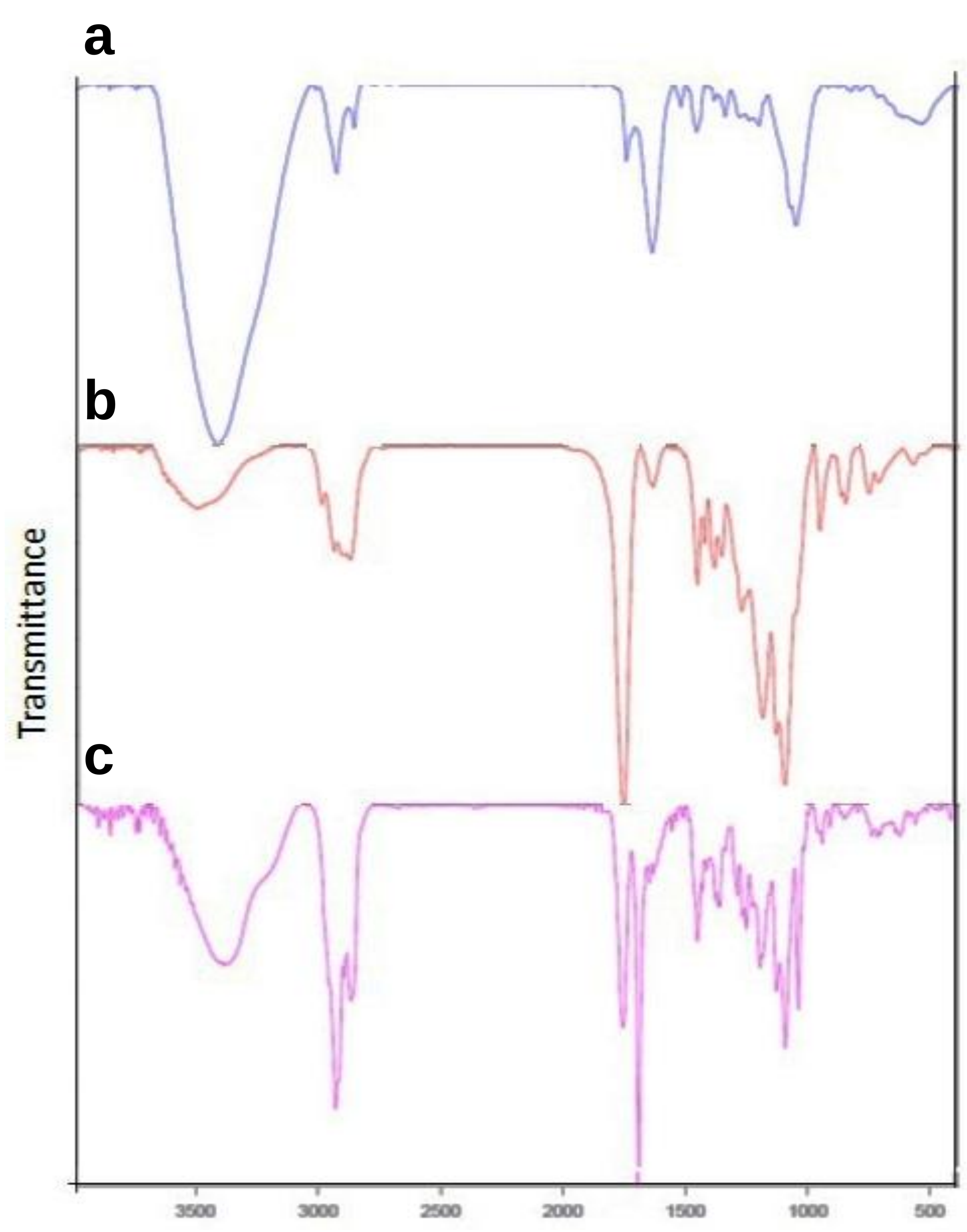

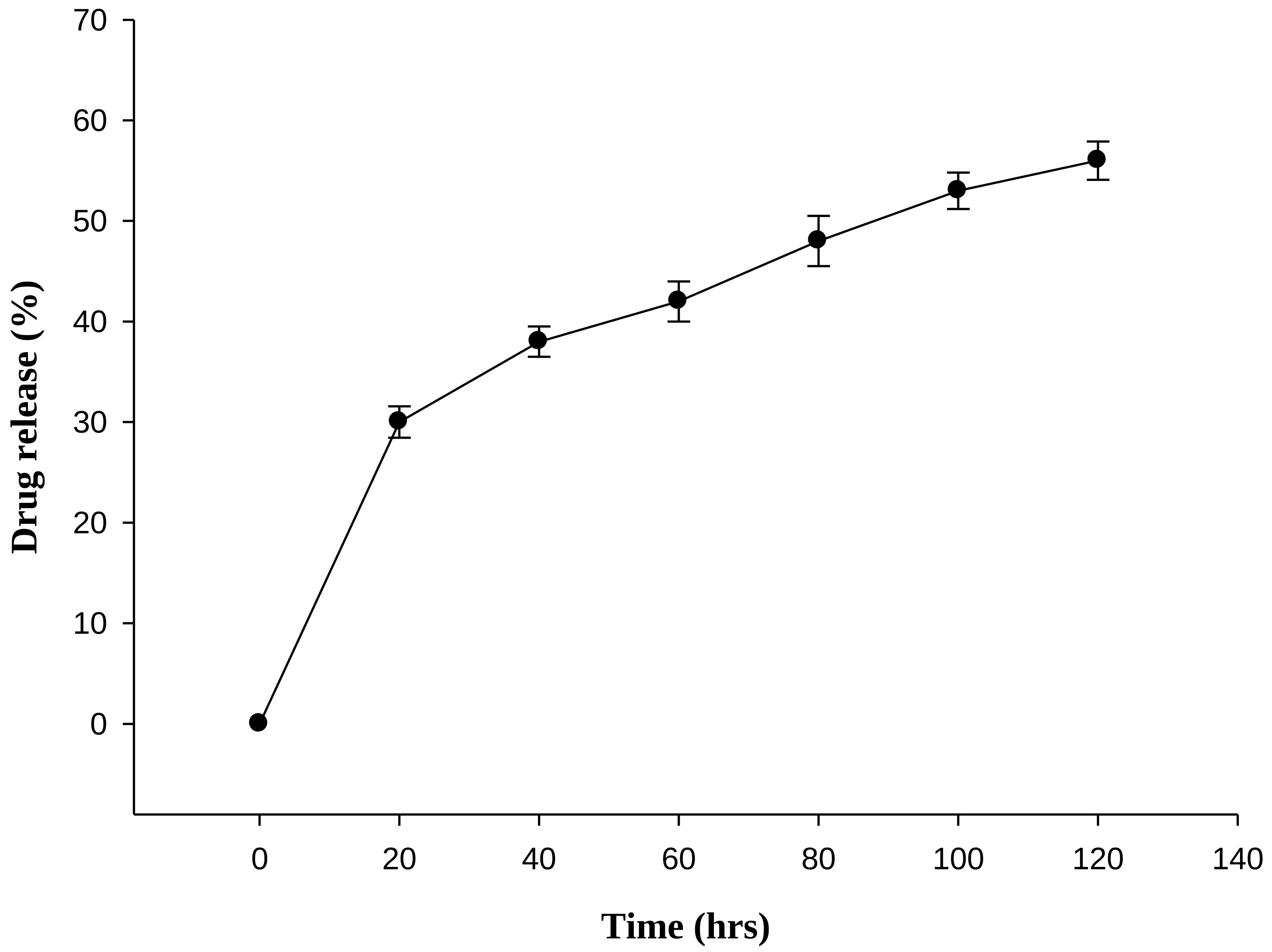

Fig. S1. FT-IR spectra of (a) anthocyanin (b) PLGA-PEG NPs (c) anthocyanin-loaded NPs

Fig. S2. *In vitro* drug release of Anthocyanin-NPs

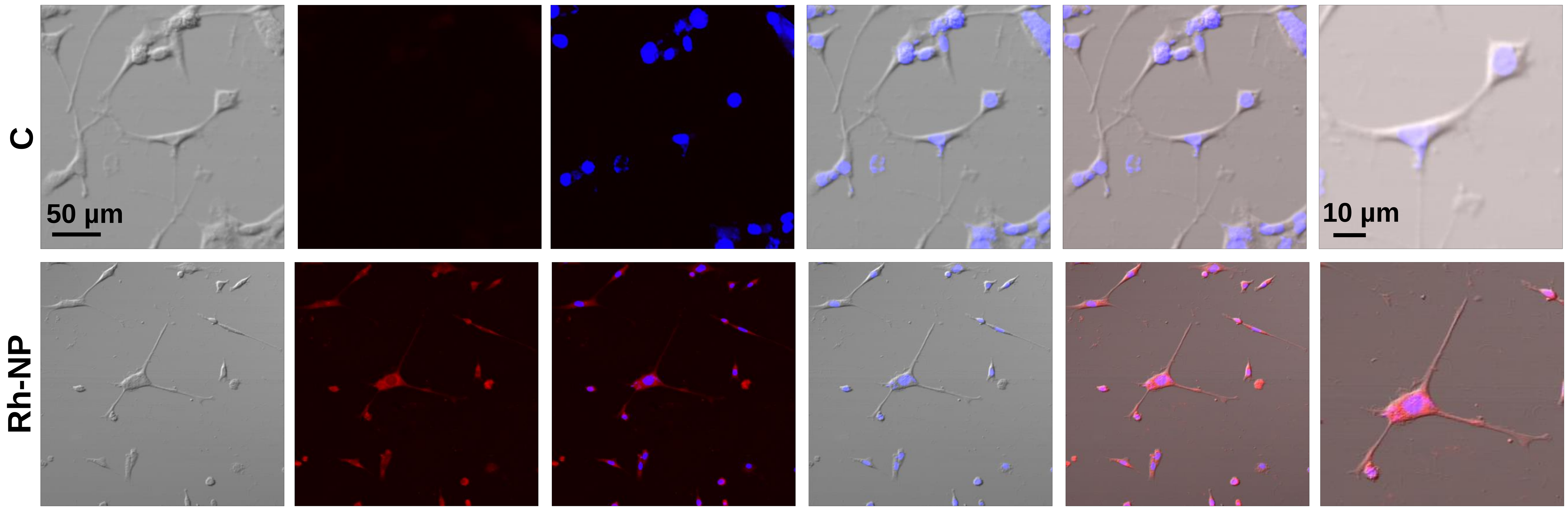

Fig. S3. Microscopic study showing the cellular internalization of the Rh-NPs
